# Exploring urban–rural inequities in older adults life expectancy: a case study in Zhejiang, China for health equity

**DOI:** 10.3389/fpubh.2025.1439857

**Published:** 2025-02-03

**Authors:** Yongguo Chen, Xiaoting Fan, Shusheng Shen, Yong Chen, Zhiwei Pan, Zixuan Chen, Haoqiang Zhong, Menglong Li

**Affiliations:** ^1^School of Civil Engineering and Architecture, Zhejiang University of Science and Technology, Hangzhou, China; ^2^Zhejiang-Singapore Joint Laboratory for Urban Renewal and Future City, Hangzhou, China

**Keywords:** the older adults, life expectancy, urban–rural inequities, health inequities, social, determinants of health

## Abstract

This study investigates the inequities in life expectancy among individuals aged 65 and above in urban and rural areas of Zhejiang Province, China, with a primary focus on promoting health equity among the older adults population. The objective is to analyze the trends and factors contributing to the urban–rural gap in life expectancy and to propose strategies for reducing this disparity. Data from the 2010 and 2020 statistical records and census data were analyzed using cohort life tables and gray correlation analysis. Results indicate an overall increase in life expectancy among the older adults, with a more pronounced improvement in rural areas, thereby narrowing the urban–rural gap from 1.53 years in 2010 to 1 year in 2020. Income inequality emerges as the primary factor influencing life expectancy, followed by educational attainment, with variations across different age groups and gender. This underscores the importance of tailored interventions that consider the specific needs of older adults individuals in diverse geographical areas and age brackets to extend life expectancy and promote health equity. By tackling these unfair differences, health equity can be ensured and the overall well-being of the older population in both urban and rural areas can be improved.

## 1 Introduction

Health equity, defined as the endeavor to reduce avoidable disparities in health and its determinants—encompassing not only healthcare but also broader factors—among groups of people characterized by varying levels of underlying social advantage or privilege (1). Societal-level inequality inherently harms the entire population (2), and these inequalities can be reflected in the life expectancy of populations in different regions. According to the data disclosed in the World Health Statistics Report (2023) published by the World Health Organization, global life expectancy jumped from 46.5 to about 73.0 years from 1950 to 2019. It is projected that by 2048, the average global life expectancy is expected to reach 77.0 years (3). China’s National Health and Wellness Commission released the Healthy China Initiative (2019–2030), which explicitly sets specific target values for China’s life expectancy to reach 77.7 years in 2022, and aims to reach 79.0 years by 2030 (4). Throughout the Yangtze River Delta (YRD) region, many health policies have also been proposed individually. By analyzing Zhejiang’s practices in the context of the YRD, we can gain insight into the factors affecting life expectancy and identify opportunities for collaboration and improvement. In Zhejiang Province, particular attention has been paid to reducing the gap between urban and rural life expectancy, as reflected in the province’s specific development strategies. Guided by the “Eight-eight Strategy,” which clearly states that the goal of Zhejiang Province is to achieve a life expectancy of 82.4 years by 2027 (5), this strategy emphasizes urban–rural integration, by investing in rural infrastructure, medical facilities and education, in order to promote the sustainable development of society. It can be seen that, along with the policy orientation, the attention from society and academia towards the measurement indicators and influencing factors of healthy life expectancy has also increased (6).

Life expectancy serves as a crucial indicator of population health and social development, and it has witnessed a general increase in recent years due to advancements in medical technology, living conditions, and public health policies. However, this increase is not solely linear; rather, it is influenced by a variety of factors leading to diverse outcomes. Despite the overall increasing trend in global life expectancy, significant inequalities persist among different countries, regions, and races (7, 8), largely influenced by disparate living habits, cultural practices, and socioeconomic status (9). Furthermore, the unique characteristics of groups affected by social determinants of health, including territorial factors, gender differences and disability status, significantly shape their access to healthcare resources and quality of life, thereby influencing their life expectancy in profound ways. For instance, in certain less developed regions, life expectancy remains lower due to issues such as resource scarcity and poverty (10). Moreover, significant disparities in life expectancy exist between urban and rural areas (11, 12). Rural residents tend to have lower life expectancy than urban residents due to relatively inadequate social and medical conditions and differences in living environments (13). Nevertheless, the urban–rural gap is gradually narrowing with the enhancement of economic and medical conditions in rural areas (14, 15). Gender differences should not be overlooked. Generally, female life expectancy exceeds that of males in most countries and regions (16), except for a few of the world’s poorest nations (17). Nonetheless, gender differences are somewhat mitigated by a combination of various factors, potentially resulting in subsequent alterations in their correlation with life expectancy (18). In addition, the analysis showed significant differences in life expectancy based on disability, with years lost to disability being a linear function of life expectancy at birth (19). Older adults individuals face an increasing risk of disability (20). Each of these factors has a significant impact on social groups, and in more detail, territory differences that generate geographic and cultural differences in access to health services, gender differences that may affect health outcomes from an innate perspective, and disabilities that can affect quality of life by limiting mobility and social participation, all further exacerbate these inequalities. Presently, greater research emphasis is placed on investigating the specific factors influencing life expectancy. Particularly, socio-public factors such as socio-economic development, health expenditures, and health services have been identified as significant determinants affecting life expectancy (21–24). It is noteworthy that environmental factors, including carbon emissions and energy, have garnered considerable attention (25–27). Additionally, personal factors such as income level, education level, and health behaviors are deemed to be closely intertwined with life expectancy (28–31). Nonetheless, there remains a dearth of current research on the precise degree of correlation between these social factors and life expectancy among the older adults population, delineating primary and secondary factors. Considering that social roles are in a state of continual flux, the influence of these factors on the life expectancy of the older adults population may adopt a more intricate and multifaceted stance. Hence, there is an urgent need for further in-depth studies and research.

With societal progress and advancements in medical technology, the current focus on increasing population life expectancy has shifted towards reducing mortality rates among the older adults (32). Zhejiang Province is among the provinces experiencing relatively high levels of aging. Additionally, Zhejiang Province experiences the phenomenon of “urban–rural inversion” in aging. According to the results of the seventh population census, in 2020, the proportion of individuals aged 65 and above in urban areas of Zhejiang Province was 10.32%, compared to 20.90% in rural areas, reflecting a difference of approximately 10.6 percentage points (33). The degree of aging in rural areas is much higher than that in urban areas, and some studies predict that this phenomenon will further intensify (34). In contrast to inverted ageing, the coexistence of urban and rural systems has resulted in urban residents generally having better access to health care (35). However, differences in health outcomes between urban and rural residents are more likely to be due to the social determinants of health rather than just the availability of health care services. In the current society, resource allocation is usually more skewed towards urban areas, these phenomena appear to be inevitable consequences of urbanization, which also results in rural residents often facing significant challenges due to reasons such as lower socioeconomic status, limited education, and inadequate social support networks. This suggests that the health needs of a significant portion of the rural older adults population may have been overlooked to some extent, highlighting a substantial inequity between urban and rural regions (36), posing significant challenges to societal sustainability. Thus, investigating inequities in life expectancy and its determinants among urban and rural older adults populations in Zhejiang Province can offer a scientific foundation for the government to devise more precise geriatric health policies. This, in turn, can help bridge the urban–rural gap and foster social equity, ultimately promoting sustainable societal development in Zhejiang Province.

The main objectives of this study are as follows: (1) Constructing a life table and measuring life expectancy using data from the Sixth and Seventh Population Census of Zhejiang Province, the database of the Zhejiang Provincial Health and Health Commission, and data published by the Zhejiang Provincial Bureau of Statistics; (2) Calculating the degree of correlation between life expectancy and the specific characteristics of the population in different regions (urban and rural) that are related to differences in life expectancy, using a gray correlation analysis method. (3) Analyzing changes in life expectancy and its influencing factors based on the study results for the years 2010 and 2020, including horizontal analysis of time trends and vertical comparison of urban and rural geographic differences; (4) Analyze key gender differences in life expectancy factors, to inform ageing health policies promoting social equity and sustainable development.

## 2 Materials and methods

### 2.1 Study design

**Figure 1:**
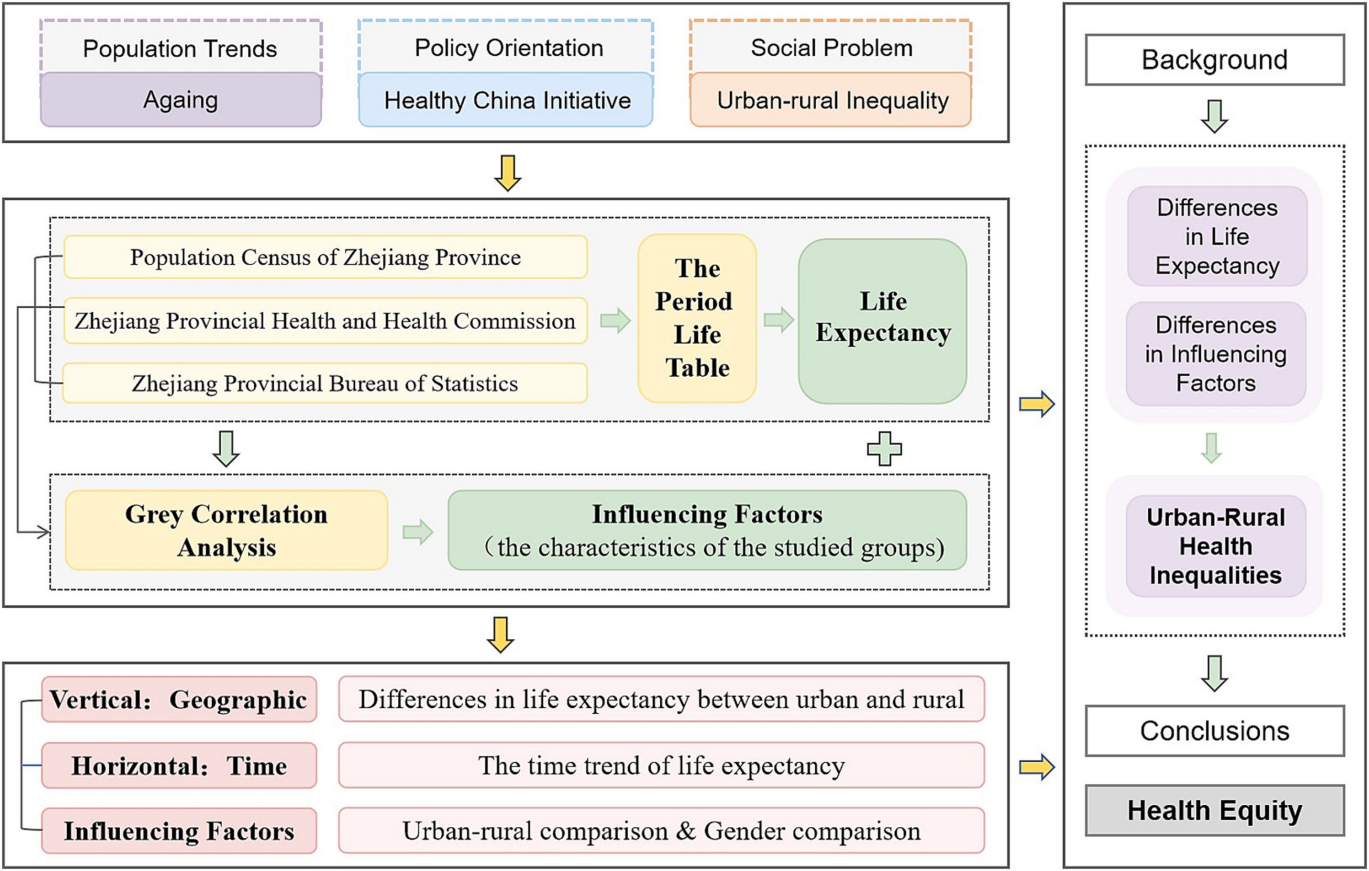
Study design.

This section outlines the detailed plan and methodology employed in the study. It begins with a visual representation of the study design, specifically a descriptive study, in the form of a mind map. This mind map provides a concise overview of the research process, including the background of the study, data sources, research methodology and analysis, and steps for the purpose of the study. As shown in Figure 1.

### 2.2 Materials

The calculation of life expectancy in this study requires data on the survival and death of the older adults population aged 65 years or older, categorized by age group and region. The study sample consists of individuals from Zhejiang Province, China, who were aged 65 and above during the periods covered by the sixth population census (2010) and the seventh population census (2020). These data are primarily sourced from these two censuses and are calibrated using information provided by the Zhejiang Provincial Bureau of Statistics (SPBS) and the Zhejiang Provincial Center for Disease Control and Prevention (CDC), as detailed in Table 1. Additionally, this study adhered to international standards by defining the age of the older adults population as 65 years old to ensure result generalizability and comparability. To maintain data consistency, urban administrative divisions comprising “city” and “town” were consolidated into the urban category, while rural areas retained their original administrative divisions. This approach facilitates a more accurate analysis of disparities between urban and rural areas and their impact on older adults population life expectancy.

**Table 1:** Survivors and deaths of the older adults population aged 65 and above in urban and rural areas of Zhejiang Province in 2010 and 2020 (persons).

Year	Area		Life expectancy of age groups (persons)					
			65–69	70–74	75–79	80–84	85–89	90—
2010	Urban	$h_{x}$ ^a^	744,586	611,953	531,544	305,261	135,766	43,830
	Rural	$d_{x}$ ^b^	7,526	11,978	18,488	20,854	16,401	9,437
2020	Urban	$h_{x}$	776,071	708,890	635,604	374,445	165,058	48,667
	Rural	$d_{x}$	12,183	20,167	30,070	33,081	25,033	13,288

a ￼=number of survivors at age x.

b ￼=number of death at age x.

The data on influencing factors required for this study primarily originate from the sixth population census of Zhejiang Province in 2010, the seventh population census in 2020, the Zhejiang Provincial Bureau of Statistics, and the statistical yearbook. These variables encompass three levels: social, economic, and personal. These include social welfare, income, education, health status, gender, and whether the older adults lives alone. Specifically, the social dimension includes “social welfare,” which in our study refers to social security programs such as retirement pensions, unemployment insurance, and minimum living allowances, measured as a proportion of the total population. The economic dimension encompasses “income” calculated as the average income of the study population across various age groups. The personal dimension spans “education” measured by the duration of educational attainment. The “health status” primarily referring to an individual’s capacity to live independently and categorized into “healthy” and “unhealthy” based on survey data from the censuses. And “whether the older adults lives alone” determined by the household population size in the survey. These factors, which are well-established in previous studies (21–31) as being correlated with life expectancy, were selected for analysis to determine the specific degree of association with life expectancy and identify primary and secondary factors.

### 2.3 Methods

#### 2.3.1 The period life table

This study employed the construction of period life tables to measure life expectancy. The life table is a statistical model compiled based on the probability of death at each age, noted for its ease of production, intuitiveness, and simplicity (37). The life table examines the survival of individuals born in the same period at different ages (38). It can be divided into the Cohort Life Table and the Period Life Table. The latter conducts a cross-sectional analysis of the life process by assuming the current age-specific probability of death throughout life to measure life expectancy. This latter approach calculates the average number of years of survival for the same group of people, considering the current probability of dying at the current age (5). In this study, 65 years of age were utilized as the starting point for the older adults age group, with a subsequent age interval of 5 years. The final age group comprised individuals aged >90 years, while the remaining age groups were divided based on a 5-year criterion. Compilation followed the methodology outlined in prior studies (5, 39).

The number of survivors $h_{x}$ (where x represents age) and deaths $d_{x}$ by age group, as per the data published by the respective statistical offices, are displayed in Table 1. The fundamental data for computing the period life table is the mortality rate $m_{x}$ for each age group, serving as the basis for deriving all other indicators (40). This ratio, calculated by the formula, represents the number of deaths relative to the number of survivors:

$$m_{x}=d_{x}/h_{x}$$

The probability of death $q_{x}$ represents the likelihood that individuals who have reached age x will die before reaching age x+n , assuming a uniform distribution of deaths within the age interval. This probability is calculated using this formula:

$$q_{x}=\frac{nd_{x}}{h_{x}}=\left(2\times n\times m_{x}\right)/\left(2+n\times m_{x}\right)$$

The number of survivors $l_{x}$ refers to the count of individuals within each age group who have survived, typically beginning with the first group $m_{x}=10,000$ :

$$l_{x}=l_{x-1}-D_{x}$$

The number of life table deaths $D_{x}$ represents the individuals in each age group expected to die among the number of survivors $l_{x}$ , based on the prevailing probability of death $q_{x}$ . It is calculated using the formula:

$$D_{x}=q_{x}\times l_{x}$$

Survivor years $L_{x}$ represent the cumulative number of years survived by each age group, with the number of survivors $l_{x}$ aged 65 serving as the baseline:

$$L_{x}=\{\begin{array}{c} \left(l_{x}+l_{x+n}\right)\times n/2\\ l_{x}/m_{x},x=\mathrm{Highest}\;\mathrm{age}\;\mathrm{group} \end{array}$$

Total survivor years $T_{x}$ represent the cumulative survivor years from age x to the maximum age, where the maximum age is denoted as ω :

$$T_{x}=\overset{\omega }{\underset{x}{\sum }}L_{x},L_{\omega }$$

Finally, calculating life expectancy $E_{x}$ :

$$E_{x}=T_{x}/l_{x}$$

#### 2.3.2 Grey correlation analysis

Gray correlation analysis is a scientific method used for quantitatively describing and comparing the developmental dynamics of system changes. Its primary purpose is to calculate the correlation degree of each factor and identify the key factors influencing the independent variable based on ranking (41). Following the steps of gray correlation analysis, the calculated results are categorized into different years, regions, and age groups, corresponding to the gray correlation. Based on the correlation degree calculated from the aforementioned grouping, which indicates the correlation strength of each factor with the reference variable, the variables closely related to the reference variable are determined. Additionally, the magnitude of the data resulting from the relationship between each factor and the reference variable was ranked to precisely convey the influence of each factor on life expectancy, facilitating the identification of primary and secondary factors driving changes in the dependent variable.

In this paper, calculations were conducted following the methods outlined by Zhao (42). Firstly, the analytical series were identified, and the selected variables were categorized into one dependent factor and several independent factors. The series containing the dependent variable served as the reference series $\left\{x_{0}\right\}$ , while those containing the independent variables constituted the comparative series $\left\{x_{m}\right\}$ . Secondly, the variables’ series were normalized, and the initial data point of each series was used to normalize the subsequent data points, thereby transforming them into comparable data series. Subsequently, the difference series was derived using the following formula:

$$\Delta m\left(n\right)=\left|x_{0}\left(n\right)-x_{m}\left(n\right)\right|$$

Maximum Difference:

$$A=\max\left(m\right)\max\left(n\right)\Delta m\left(n\right)=\max\left(m\right)\max\left(n\right)\left|x_{0}\left(n\right)-x_{m}\left(n\right)\right|$$

Minimum Difference:

$$B=\min\left(m\right)\min\left(n\right)\Delta m\left(n\right)=\min\left(m\right)\min\left(n\right)\left|x_{0}\left(n\right)-x_{m}\left(n\right)\right|$$

Next, the gray correlation coefficient is calculated by dividing the sum of A and B by the sum of $\Delta m\left(n\right)$ and A :

$$\eta_{m}\left(n\right)=\frac{\min\left(m\right)\min\left(n\right)\left|\begin{array}{l} x_{0}\left(n\right)-\\ x_{m}\left(n\right) \end{array}\right|+\mathrm{\phi Max}\left(m\right)\max\left(n\right)\left|\begin{array}{l} x_{0}\left(n\right)-\\ x_{m}\left(n\right) \end{array}\right|}{\left|x_{0}\left(n\right)-x_{m}\left(n\right)\right|+\mathrm{\phi Max}\left(m\right)\max\left(n\right)\left|x_{0}\left(n\right)-x_{m}\left(n\right)\right|}$$

Finally, the gray correlation is obtained by averaging the gray correlation coefficients of each column:

$$S_{m}=\frac{1}{t}\overset{t}{\underset{n=1}{\sum }}\eta_{m}\left(n\right)$$

## 3 Results

### 3.1 Life expectancy

In this study, we utilized EXCEL software to prepare and compute life tables using census data, thereby deriving the life expectancy of the older adults population aged 65 and above in both urban and rural areas of Zhejiang Province for the years 2010 and 2020, as depicted in Table 2, respectively. The calculated results are stratified by various years, regions, and age groups, offering a more comprehensive depiction of life expectancy among the older adults population. Based on the life table calculation results, notable trends emerge: firstly, the life expectancy of both urban and rural older adults populations in Zhejiang Province has markedly risen over the decade spanning from 2010 to 2020, aligning with anticipated trajectories and conforming to the principles of social development and advancement. Additionally, the hierarchy of “urban older adults population > rural” in terms of life expectancy persists. This pattern is evident not only across the entirety but also within all age brackets of both urban and rural locales. Conversely, while an urban–rural disparity persists, it has notably diminished over the course of the decade, corroborating the conclusions drawn by numerous scholars (14, 15). This suggests that Zhejiang Province has made strides in mitigating the urban–rural divide and enhancing the health outcomes of the rural older adults population.

**Table 2:** Life expectancy of the older adults population aged 65 and above in urban and rural areas of Zhejiang Province in 2010 and 2020 (years).

Year	Area	Life expectancy of age groups (years)					
		65–69	70–74	75–79	80–84	85–89	90—
2010	Urban	19.71	15.60	11.95	8.75	6.33	4.64
	Rural	17.43	13.65	10.35	7.46	5.27	3.66
2020	Urban	22.08	17.75	13.75	10.24	7.57	5.54
	Rural	20.57	16.49	12.66	9.36	6.87	4.99

**Figure 2:**
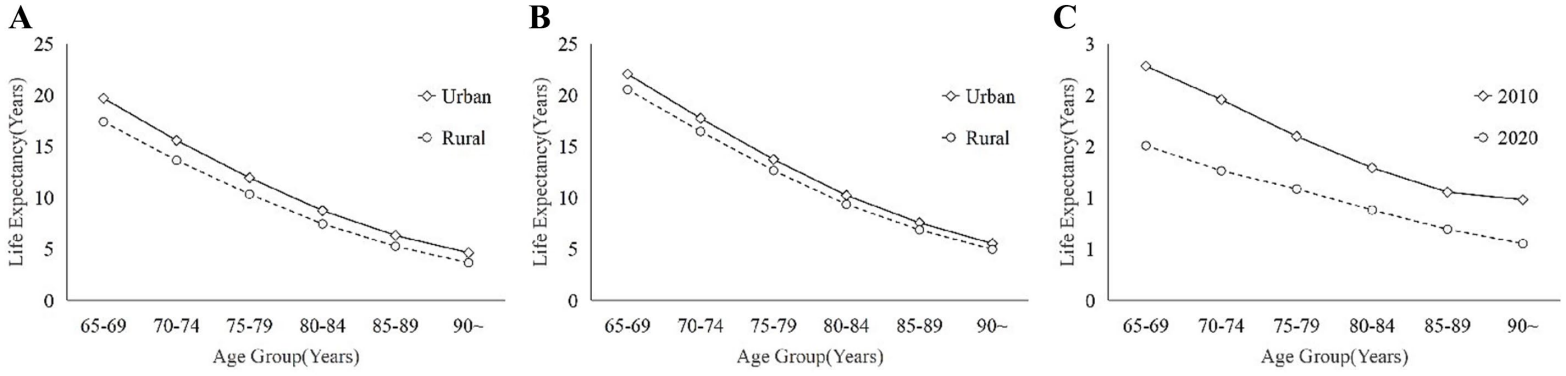
These illustrates the differences in life expectancy between urban and rural areas between different years: **(A)** 2010; **(B)** 2020; and **(C)** Urban–rural differences between 2010 and 2020.

**Figure 3:**
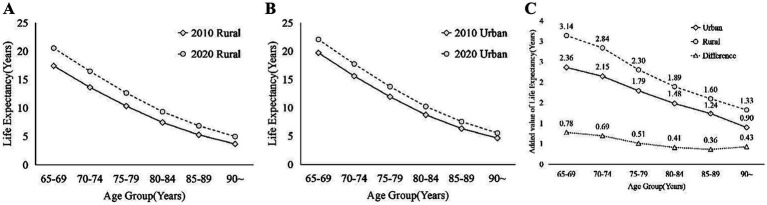
The difference of the older adults population in Zhejiang Province between 2010 and 2020 in different areas: **(A)** urban; **(B)** rural; **(C)** increase in life expectancy between 2010 and 2020.

**Figure 4:**
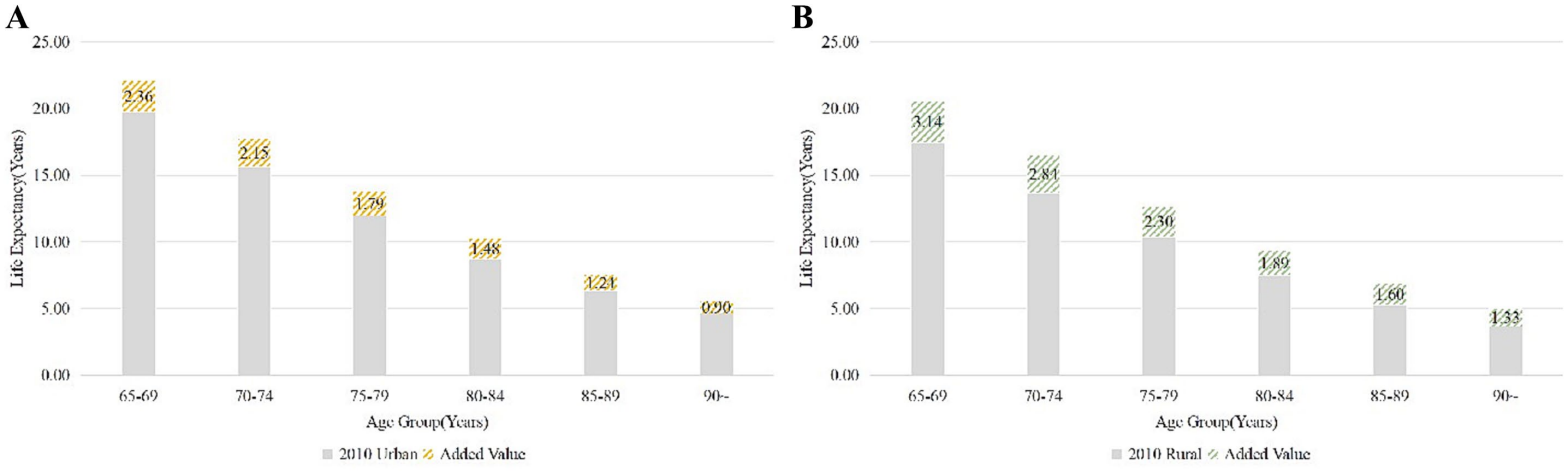
These illustrates the added value of life expectancy of urban older adults population aged 65 and above in Zhejiang Province at all age group, from 2010 to 2020: **(A)** Urban; **(B)** Rural.

In addition, in order to more visually reveal the significant differences in life expectancy that exist between urban and rural areas and over different time spans in Zhejiang Province, we have also plotted the following figures. Figure 2 shows life expectancy in 2010 and 2020, with Panel (C) highlighting the reduction in urban–rural differences over time. Figure 3 depicts changes in the older adults population, distinguishing between urban and rural areas, and indicates an increase in life expectancy from 2010 to 2020. Lastly, Figure 4 compares the added value of life expectancy for urban older adults aged 65+ across all age groups from 2010 to 2020, between urban and rural settings.

### 3.2 Factors

Correlations among various influencing factors were determined through gray correlation analysis (Table 3), and the resultant calculations were stratified across different years, regions, and age groups. A higher correlation value (closer to 1) indicates a stronger relationship with the reference variable.

**Table 3:** Correlation degree of influencing factors on life expectancy of the older adults people over 65 in all age groups in urban and rural areas of Zhejiang Province in 2010 and 2020.

Year	Area	Age Groups	Degree of factors^1^				
			Welfare	Income	Education	Health Status	Non-living alone
2010	Urban	65–69	0.840	0.916	0.935	0.920	0.764
		70–74	0.751	0.851	0.898	0.862	0.583
		75–79	0.703	0.846	0.874	0.824	0.452
		80–84	0.691	0.861	0.858	0.803	0.375
		85–89	0.699	0.894	0.848	0.792	0.338
		90-	0.713	0.914	0.848	0.813	0.333
	Rural	65–69	0.741	0.973	0.953	0.921	0.756
		70–74	0.639	0.907	0.936	0.870	0.579
		75–79	0.573	0.877	0.920	0.834	0.458
		80–84	0.557	0.868	0.901	0.819	0.385
		85–89	0.562	0.892	0.887	0.811	0.345
		90–	0.576	0.917	0.895	0.819	0.338
2020	Urban	65–69	0.869	0.912	0.929	0.923	0.863
		70–74	0.769	0.849	0.868	0.864	0.663
		75–79	0.713	0.839	0.826	0.820	0.507
		80–84	0.689	0.858	0.803	0.797	0.406
		85–89	0.674	0.883	0.788	0.789	0.348
		90-	0.677	0.911	0.784	0.802	0.341
	Rural	65–69	0.797	0.951	0.929	0.923	0.829
		70–74	0.673	0.892	0.869	0.865	0.658
		75–79	0.612	0.866	0.831	0.827	0.512
		80–84	0.614	0.866	0.814	0.807	0.418
		85–89	0.604	0.887	0.803	0.803	0.355
		90–	0.617	0.906	0.803	0.817	0.349

^1^The higher the Degree of factors (closer to 1) the stronger the correlation.

In urban areas in 2010, income, educational attainment, and health status exhibited a notably strong correlation with life expectancy. Their correlations, surpassing 0.8 across all age groups, underscore the critical significance of these factors for the urban older adults population (30). Health status also demonstrates a strong correlation, albeit slightly lower than that of education and income. Conversely, in rural areas, the 2010 data reveal notably elevated correlations between income status, educational attainment, and life expectancy, surpassing those observed in urban areas. Although the correlation for health in rural areas is relatively modest, it still exceeds 0.7. The urban–rural disparity in welfare is most pronounced, particularly among older age groups, wherein the gap notably widens with advancing age. Furthermore, the correlation of non-living alone status is consistently the lowest across all age groups in both urban and rural areas, diminishing notably with increasing age. In particular, the correlation decreases significantly among those aged 70 and over, for example from 0.764 in the 65–69 age group to 0.333 in the 90 and over age group in urban areas.

In 2020, the correlation for income in urban areas has diminished slightly but still maintains a high level exceeding 0.8. Similarly, the correlation for educational attainment experiences a slight decrease yet remains significant, indicative of the enduring impact of education on individuals’ health and socioeconomic status (29). Conversely, the correlation for health status has strengthened, persisting above the 0.9 threshold for the 65-year age group.

Table 4 presents gender comparisons of the correlation between influencing factors and the life expectancy of older adults people over 65 years old, across all age groups in both urban and rural areas of Zhejiang Province, for the years 2010 and 2020. Income stands out as the top factor for both males (M) and females (F), highlighting its crucial role. Education is significant, especially in rural areas in 2020. Health status is also key, with a slightly higher impact on males. Welfare and non-solitary status have lesser associations, gender differences in these factors’ impact are minimal.

**Table 4:** Gender comparisons of the correlation of influencing factors on life expectancy of the older adults people over 65 in all age groups in urban and rural areas of Zhejiang Province in 2010 and 2020.

Year	Area	Age groups	Degree of factors^1^									
			Welfare		Income		Education		Health status		Non-living alone	
			M^2^	F^3^	M	F	M	F	M	F	M	F
2010	Urban	65–69	0.584	0.496	0.788	0.566	0.778	0.765	0.772	0.658	0.758	0.701
		70–74	0.443	0.365	0.649	0.444	0.666	0.697	0.647	0.511	0.620	0.577
		75–79	0.364	0.337	0.626	0.466	0.588	0.682	0.574	0.444	0.543	0.514
		80–84	0.338	0.335	0.647	0.516	0.542	0.659	0.536	0.413	0.497	0.456
		85–89	0.339	0.333	0.716	0.590	0.527	0.585	0.516	0.397	0.476	0.398
		90-	0.333	0.333	0.762	0.647	0.531	0.542	0.545	0.428	0.463	0.348
	Rural	65–69	0.573	0.565	0.968	0.870	0.875	0.969	0.860	0.808	0.844	0.831
		70–74	0.444	0.467	0.888	0.669	0.814	0.984	0.778	0.708	0.745	0.741
		75–79	0.360	0.415	0.832	0.652	0.763	0.998	0.723	0.646	0.683	0.687
		80–84	0.333	0.399	0.793	0.679	0.725	0.938	0.696	0.627	0.643	0.637
		85–89	0.350	0.353	0.822	0.737	0.697	0.872	0.684	0.613	0.624	0.585
		90-	0.373	0.333	0.858	0.794	0.703	0.852	0.686	0.630	0.618	0.538
2020	Urban	65–69	0.628	0.619	0.765	0.711	0.794	0.732	0.792	0.698	0.776	0.716
		70–74	0.462	0.445	0.637	0.538	0.661	0.573	0.666	0.551	0.644	0.606
		75–79	0.394	0.368	0.625	0.501	0.578	0.500	0.588	0.470	0.562	0.552
		80–84	0.365	0.341	0.657	0.540	0.531	0.469	0.545	0.437	0.510	0.516
		85–89	0.341	0.333	0.702	0.604	0.496	0.455	0.529	0.429	0.494	0.472
		90-	0.333	0.338	0.760	0.684	0.485	0.446	0.543	0.451	0.489	0.412
	Rural	65–69	0.570	0.431	0.958	0.901	0.923	0.877	0.926	0.853	0.915	0.874
		70–74	0.540	0.427	0.907	0.780	0.861	0.782	0.869	0.758	0.846	0.807
		75–79	0.603	0.591	0.880	0.736	0.816	0.736	0.831	0.702	0.798	0.773
		80–84	0.506	0.540	0.876	0.748	0.791	0.724	0.809	0.677	0.769	0.742
		85–89	0.397	0.401	0.893	0.790	0.768	0.698	0.799	0.676	0.760	0.708
		90-	0.333	0.333	0.912	0.823	0.777	0.720	0.812	0.695	0.764	0.654

^1^ The higher the Degree of factors (closer to 1) the stronger the correlation. ^2^ M = Male. ^3^ F = Female.

## 4 Discussion

### 4.1 Differences in life expectancy between urban and rural

The life expectancy of the older adults population aged 65 and above in Zhejiang Province in 2010 by age group is shown in Figure 2A, which provides visual information on the health status and medical care level of the older adults population. It’s noteworthy that the solid line in Figure 2C delineates the disparity in life expectancy between urban and rural older adults populations. Figure 2A illustrates that the life expectancy of the older adults population in urban generally exceeds that in rural, possibly attributable to superior medical facilities, living standards, and older adults care in urban areas (43). Nevertheless, it is observed that the disparities between urban and rural areas among different age groups of the older adults population exhibit varying characteristics. Specifically, Figure 2C depicts that the urban–rural disparities in life expectancy among the older adults, from the age group of 65–69 to 85–89, exhibit a significant yet consistently diminishing trend, with the disparities tapering off in the age group of 90 and above. This trend may reflect advancements in rural areas concerning enhanced living conditions and improved medical care for the older adults (44).

Figure 2B illustrates the life expectancy of the older adults population in Zhejiang Province in 2020 for all age groups aged 65 and above, while the dotted line in Figure 2C further reveals the changes in the differences between urban and rural areas. Observing Figure 2B, it’s evident that the life expectancy of the older adults population has generally risen. However, the consistent pattern persists whereby the life expectancy of urban older adults individuals surpasses that of their rural counterparts, aligning with research on the overarching urban–rural disparity in life expectancy in China (12), and globally (11, 45, 46). Upon juxtaposing Figures 2B,C, it’s apparent that the gap in life expectancy between urban and rural areas for both younger and older adults individuals in 2020 exhibits a consistent trend of reduction and uniform narrowing in comparison to 2010.

The urban–rural disparities in life expectancy among the older adults population aged 65 and above in Zhejiang Province for 2010 and 2020 are examined using descriptive statistics based on the aforementioned data, with the findings presented in Table 5. Considering Figure 2C alongside, it’s evident that the urban–rural disparity across all age groups was greater in 2010 compared to 2020. The average urban–rural difference was 1.53 years in 2010, and 1.00 years in 2020, indicating a 33.3% reduction. Concerning disparities among age groups, the urban–rural difference demonstrates a pattern of narrowing followed by widening. Notably, the widest gap is observed in the 65–69 age group, at 0.78 years, while the narrowest disparity is found in the 85–89 age group, at 0.36 years.

**Table 5:** Descriptive statistics of changes in life expectancy between 2010 and 2020 for urban and rural older adults populations in Zhejiang Province (years).

	2010	2020	D-value
Average	1.53	1.00	0.53
Standard Deviation	0.47	0.33	0.15
Median	1.45	0.98	0.47
Max	2.28	1.51	0.78
Min	0.98	0.55	0.36
Range	1.30	0.95	0.41

### 4.2 The time trend of life expectancy

Figure 3 illustrates the life expectancy of the older adults population of all ages in urban areas (A) and rural areas (B) for 2010 and 2020 is presented below, while Figure 3C illustrates the rise in life expectancy among the older adults population in urban and rural areas of Zhejiang Province from 2010 to 2020, alongside the urban–rural disparity. Overall, it’s evident that the life expectancy of the older adults population of all ages has increased in both urban and rural areas between 2010 and 2020. This increase is closely associated with ongoing socio-economic development, advancements in medical technology, and enhancements in public health services (44).

In urban areas, while the growth in life expectancy of the older adults population is not as significant as in rural areas, there is a noticeable increase across all age groups, as shown in Figure 4A. In comparison to 2010, the most substantial rise in life expectancy in 2020 was observed in urban areas among the 65–69 age group, with an increase of approximately 2.36 years, whereas the smallest increase was noted among individuals aged 90 years and older, at around 0.90 years. An analysis of the rise in life expectancy across different age groups indicates a tendency for the increase to decelerate with advancing age.

Similarly, in rural areas, life expectancy shows an upward trend across all ages from 2010 to 2020, as shown in Figure 4B. Although it remains higher among the urban older adults population compared to rural areas. Additionally, Figure 3C illustrates that the increase in life expectancy is more notable among the older adults population compared to urban areas. The age groups exhibit distinct trends, with the most substantial increase in life expectancy observed in 2020 among the older adults population aged 65–69 years in rural areas compared to 2010, showing an increase of over 3 years to 3.14 years. The smallest increase in life expectancy is for those aged 90 years and above, with an increase of about 1.33 years. An examination of the rise in life expectancy across different ages reveals a tendency for fluctuation and deceleration with advancing age.

Regarding the disparities between urban and rural areas in the increase of life expectancy, as depicted in the calculation results, as shown in Figure 3C. These differences exhibit an initial narrowing followed by widening with age. Particularly, the highest disparity in life expectancy increase occurs in the 65–69 age group, reaching 0.76 years. Subsequently, from the 85–89 age group onwards, this difference progressively diminishes to 0.34 years, before ascending to 0.39 years in the 90 and above age group. Table 6 presents descriptive statistics concerning the increase in life expectancy among the older adults population aged 65 and above in urban and rural areas of Zhejiang Province in 2010 and 2020, respectively. Analysis of Table 5 indicates that over a decade of development, life expectancy among the older adults population in urban areas rose by an average of 1.65 years, compared to a 2.18-year increase in rural areas. This difference signifies a substantially greater increase in life expectancy in rural areas, the gap between urban and rural areas is gradually narrowing, consistent with findings from established studies (14, 15). This trend can be attributed to the equitable allocation of medical resources, enhancements in the social security system, and ongoing improvements in the living environment (47). The cumulative impact of these favorable factors enabled rural older adults individuals to access more timely and efficient medical services, alleviate financial burdens, enhance living conditions, and adopt healthier lifestyles, consequently enhancing their quality of life and life expectancy (28, 30). This trend underscores the notable achievements of Zhejiang Province in fostering coordinated urban–rural development and enhancing the well-being of the older adults.

**Table 6:** Descriptive statistics of life expectancy of older adults population in Zhejiang Province between urban and rural (years).

	Urban	Rural
Average	1.65	2.18
Standard deviation	0.51	0.65
Median	1.64	2.10
Max	2.36	3.14
Min	0.90	1.33
Range	1.47	1.81

### 4.3 Urban–rural comparison of influencing factors in 2010, 2020

**Figure 5:**
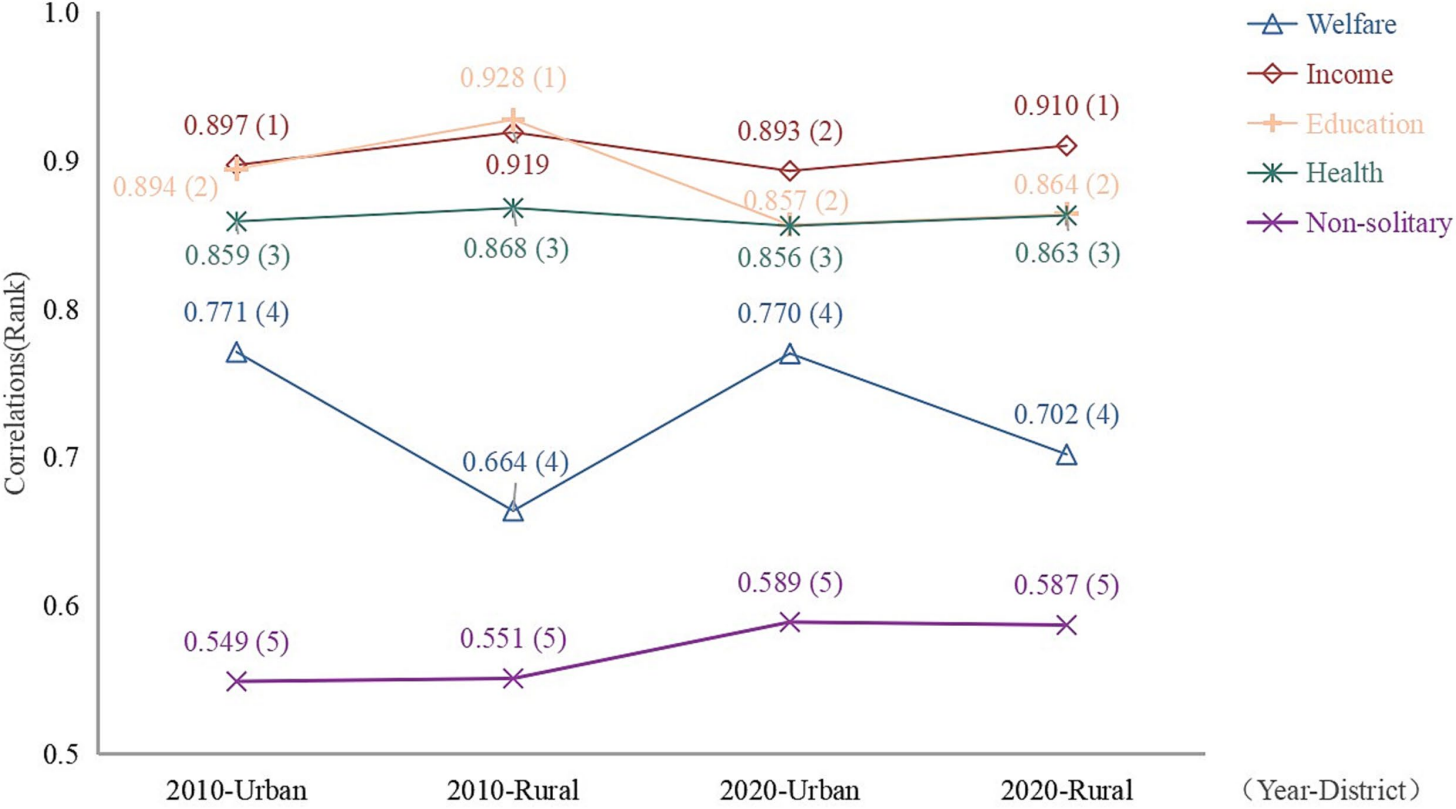
Ranking of the average value of the correlation between urban and rural elements in Zhejiang Province in 2010 and 2020.

Utilizing the correlations calculated from the aforementioned groupings, major and minor factors are ranked to provide a more comprehensible overview (Figure 5). Comparative analysis of correlation values between 2010 and 2020 enables an assessment of the influence of these factors across various age groups, urban and rural settings, and temporal changes, facilitating an exploration of urban–rural inequities.

#### 4.3.1 Welfare

From the results, this factor consistently falls within the range of 0.6 to 0.8, holding the fourth position in the rankings (Figure 5). Nonetheless, concerning specific correlation values, it does not exert an identical degree of influence on the life expectancy of the older adults population across different years and regions. The public welfare system may contribute to divergent mortality rates (48). In 2010, welfare correlation values were higher in urban areas compared to rural areas, indicating a stronger association between welfare and life expectancy among older adults population in urban settings. This phenomenon may be attributed to the superior social security system in urban areas, where older adults population are more reliant on welfare (49). In 2020, the welfare correlation experiences a slight decline in urban areas but registers a significant increase in rural areas, possibly attributable to the restructuring of social security policies in rural regions (50). In the 1950s, China implemented a free public healthcare program in urban areas, specifically targeting public sector employees and their family members, as part of a broader welfare system that aimed to provide comprehensive support to its citizens (51). The healthcare program was one aspect of the welfare system, which also included social insurance, unemployment benefits, pensions, and other forms of social protection. Notably, in 2016, China took a significant step towards achieving greater equity by extending welfare benefits, including healthcare, to rural residents on par with their urban counterparts (52). However, despite these efforts, the correlation between welfare protection and social security indicators remains higher in urban areas compared to rural areas, as evidenced by data from both 2010 and 2020 (53). This observation underscores the need for continued efforts to address the relatively low level of welfare protection in rural areas, ensuring that all citizens, regardless of their location, have access to comprehensive social protection and healthcare services.

#### 4.3.2 Income

Based on the calculation results, this factor emerges as the primary influencer of life expectancy among the older adults population in both urban and rural areas, except for rural areas in 2010 (Figure 5), aligning with previous research (54). A study pointed out that the evidence linking income inequality to population health status has primarily been observed at a broad geographic scale (55), a phenomenon likely observable in the provincial context of this study. In 2010, the urban older adults exhibited a strong correlation with income, suggesting that income played a pivotal role in meeting their fundamental needs and exerting a significant influence on life expectancy (56). Nevertheless, as society rapidly advances and progresses, the variety and complexity of factors impacting life expectancy are expanding and evolving. Amidst the intricate interplay and moderating influences of these factors (30), the correlation between income and life expectancy exhibits a subtle downward trajectory. This transition mirrors the diverse repercussions of social advancement and may indicate a more intricate and uncertain trajectory for the relationship between life expectancy and income in the future (36). The comparatively lower income correlation in rural areas in 2010 could stem from the relatively underdeveloped economic conditions in rural regions, a phenomenon observed in a study conducted in South Korea, which highlighted the likelihood of rural residents experiencing lower socio-economic status and enduring multidimensional poverty compared to their urban counterparts (57). Nonetheless, by 2020, there is a significant increase in income correlation alongside economic growth and rising income levels among rural residents. However, despite the 2020 increase, income correlation in rural areas remains lower than in urban areas due to the economic disparity between urban and rural regions.

#### 4.3.3 Education

Based on the findings, it seems that educational attainment plays a secondary role in influencing life expectancy among both urban and rural older adults populations (Figure 5), consistent with established research indicating its significance (58). Nevertheless, it’s noteworthy that the correlation was notably higher in 2010 than in 2020 for both urban and rural areas, and the disparity between urban and rural correlations was more pronounced in 2010 than in 2020. This shift may be attributed to the increased emphasis on and promotion of basic education for the older adults population in Zhejiang Province in recent years, resulting in a general improvement in education levels among both urban and rural older adults populations, particularly in rural areas (34). Consequently, this has reduced the disparity in educational attainment between urban and rural older adults populations and has fostered greater homogeneity within the population, resulting in a slight decline in the level of association. Overall, the degree of educational association remains substantial in both urban and rural areas in both 2010 and 2020. Notably, in rural areas in 2010, the correlation between educational attainment and life expectancy reached as high as 0.929. This may be due to the relative lack of educational resources in rural areas in 2010, which has led to a low emphasis on knowledge. Similar conclusions were reached in a study of healthy life expectancy among the older adults in China, which revealed through a careful social stratification analysis that only a minority of groups in rural areas had good access to education, further exacerbating inequalities in educational attainment among the population (56). These findings underscore the crucial role of education in shaping the life expectancy of the older adults population, as education levels influence individuals’ career paths, income levels, and social standing (59), enhance cognitive abilities and decision-making skills, consequently promoting health and positively impacting life expectancy (60).

#### 4.3.4 Health status

Health status consistently occupies a mid-level position (Figure 5), with its correlation to life expectancy exhibiting a similar pattern across different years in both urban and rural areas. This is likely attributable to Zhejiang Province’s status as one of China’s most developed provinces, prioritizing the health status of its residents and ensuring equitable distribution of medical resources between urban and rural areas. To be specific, in both 2010 and 2020, rural areas exhibit slightly higher health status correlations compared to urban areas. This reflects the fact that urban areas are relatively balanced in terms of healthcare resources and services, while rural areas are slightly less well-equipped in this regard (61). Nonetheless, it’s noteworthy that the correlation decreased in 2020 compared to 2010. This could be attributed to ongoing advancements in medical infrastructure in recent years, coupled with increased government investment in the healthcare sector. As medical resources continue to improve and optimize, the risk of reduced life expectancy among the urban and rural older adults populations due to inadequate healthcare resources is gradually diminishing, prompting a greater focus on healthy living practices (62). This clearly demonstrates that Zhejiang Province’s initiatives to enhance healthcare and boost investment in health are yielding favorable outcomes, ensuring robust health and life expectancy among both urban and rural older adults populations.

#### 4.3.5 Residential status: non-living alone

The correlation between non-living alone and life expectancy appears relatively low in both urban and rural areas, ranking at the bottom of the list (Figure 5). This suggests that the mode of residence may have a relatively minor impact on the life expectancy of the older adults population, or its influence is less significant compared to other factors. On the one hand, with urbanization accelerating, the older adults population in urban areas may prefer independent living or residing in care facilities; on the other hand, the older adults population in rural regions may adhere to traditional living arrangements with family members due to entrenched family values and customs. The companionship and care provided by family members play an irreplaceable and crucial role in fulfilling the emotional needs of the older adults population (34). Consequently, the disparity in non-living alone associations between urban and rural areas may fluctuate between 2010 and 2020. This trend hinges on shifts in social structures, familial dynamics, and residential preferences across diverse regions, often constrained by individuals’ abilities and opportunities to make choices (63). However, by 2020, the correlation with non-living alone had increased in both urban and rural areas, particularly in the former, resulting in a slight widening of the urban–rural disparity. This could be attributed to a rise in non-living alone arrangements accompanying shifts in family structures and increased empty-nesting in rural areas (64) (see Figure 5).

### 4.4 Gender comparison of influencing factors in different areas and years

**Figure 6:**
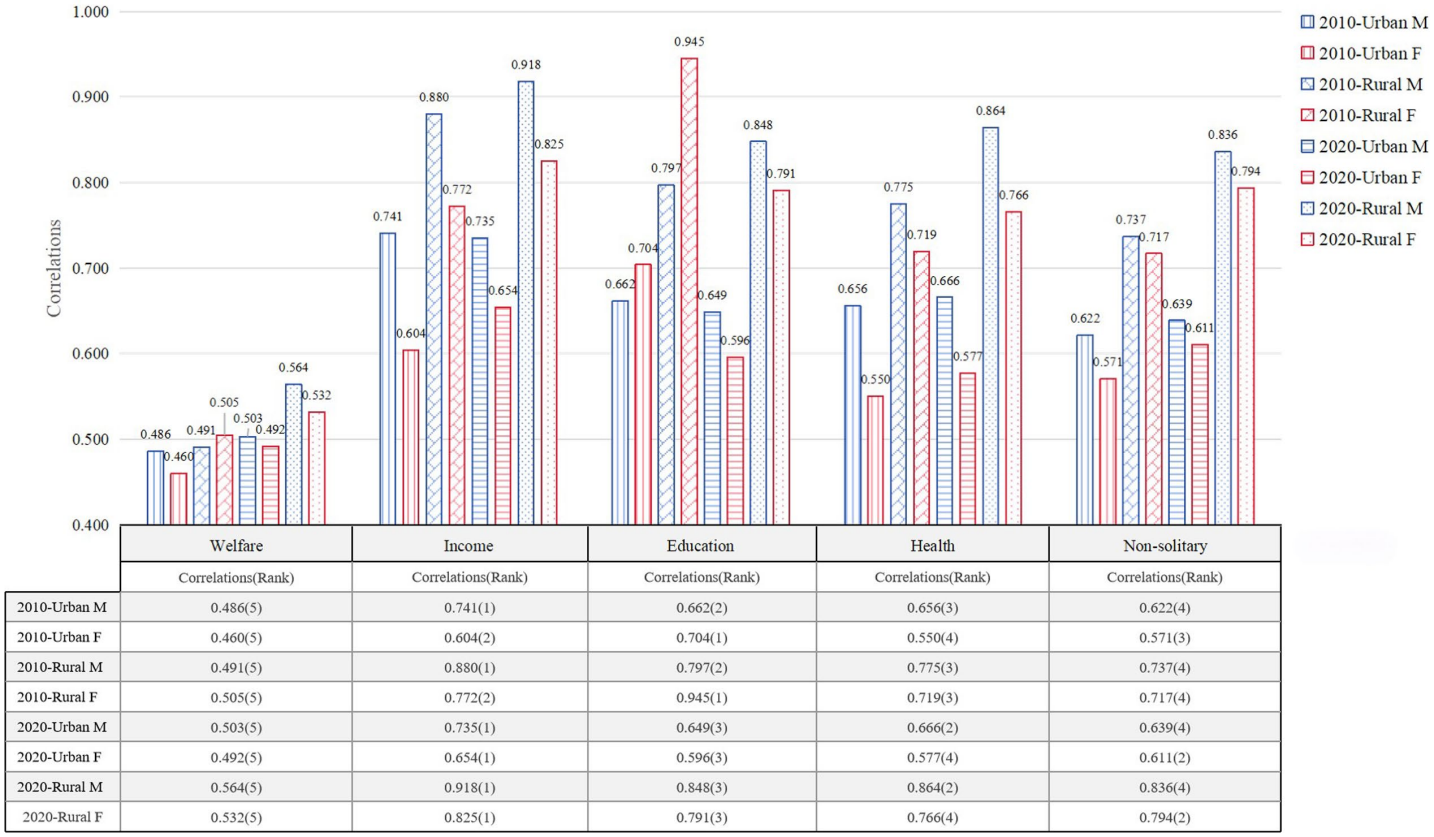
Gender comparison of influencing factors on life expectancy for older adults people over 65 in Zhejiang Province.

Figure 6 shows the ranks and correlations of factors influencing life expectancy in Zhejiang Province for people over 65 years of age in different years (2010 and 2020) and in different regions (urban and rural), with comparisons for male and female, respectively.

From the results, it is clear that the welfare factor, both urban and rural, ranked lower in terms of association in both 2010 and 2020, with little gender difference. In contrast, the income factor has a significant impact on life expectancy in 2010 and 2020 for both males and females, ranking first in most cases in both urban and rural areas. For men in particular, the association is tops in both cases. At the same time, the education factor is also an important influence on life expectancy. For males, the effect is more pronounced in rural areas, probably because urban males usually have more resources and opportunities to compensate to some extent for the lack of education level. For females, the impact of education is more pervasive. Education has a significant impact on female life expectancy in both urban and rural areas. Meanwhile, health status does not have a strong impact, with all of its correlations ranked in the middle, and overall the gender differences are not significant. Residential status (non-living alone) ranks lower in the table, but also increases. This suggests that socializing or being accompanied has a positive impact on life expectancy in old age.

## 5 Conclusion

From the life table calculations, it is evident that the life expectancy of the older adults population in Zhejiang Province is on the rise, with a more pronounced increase observed in rural areas compared to urban areas, leading to a narrowing gap between the two regions. Nonetheless, rural areas still lag behind urban areas in terms of development, posing significant challenges to achieving sustainable societal progress. The findings from the gray correlation analysis indicate that economic income disparity serves as the primary determinant, with educational attainment acting as a secondary factor. Nonetheless, the correlation of each factor varies across different regions and age groups within the older adults population. Tackling these unfair differences is crucial not only for promoting social equity but also for fostering sustainable development, ensuring the well-being of current and future generations.

Consequently, targeted interventions for the older adults should be formulated on the basis of the data collected on various influencing factors and in the light of practical realities. For example, Zhejiang Province, at a press conference on the twentieth anniversary of the implementation of the “Eight-Eight Strategy,” pointed to the highlight of “digital health care,” which is aimed at building a new system of health care that is more convenient and more accessible in order to better serve all residents. However, it remains to be seen whether this initiative will be extended to rural areas to ensure that rural residents have access to the same medical services as their urban counterparts. In addition, in addition to external help, self-health awareness of the older adults is also very important, according to the research in this paper, it is known that the level of education is one of the main factors affecting life expectancy. It is suggested that geriatric education needs to be strengthened, especially in rural areas, to increase the awareness of health issues among the older adults, which enables them to better monitor their health status. Furthermore, given the high correlation between socioeconomic status and life expectancy identified in this study, policies addressing socioeconomic disparities should also be prioritized. This may involve providing financial support and access to social services for economically disadvantaged individuals and families, especially in rural areas. In summary, these initiatives also require the concerted efforts of the Government and all sectors of society in an effort to reduce urban–rural inequities.

## Data Availability

The original contributions presented in the study are included in the article/supplementary material, further inquiries can be directed to the corresponding author.
